# Treatment Outcomes of Full Pulpotomy as an Alternative to Tooth Extraction in Molars with Hyperplastic/Irreversible Pulpitis: A Case Report 

**DOI:** 10.22037/iej.2017.51

**Published:** 2017

**Authors:** Saeed Asgary, Prashant Verma, Ali Nosrat

**Affiliations:** a * Iranian Center For Endodontic Research, Research Institute of Dental Sciences, Dental School, Shahid Beheshti University of Medical Sciences, Tehran, Iran*; b * Division of Endodontics, School of Dentistry, University of Maryland Baltimore, Baltimore, Maryland, USA*

**Keywords:** Calcium-Enriched Mixture, Hyperplastic Pulpitis, Irreversible Pulpitis, Mineral Trioxide Aggregate, Permanent Teeth, Pulp Polyp, Pulpotomy, Vital Pulp Therapy

## Abstract

Root canal therapy (RCT) is a common and successful treatment for irreversible pulpitis due to carious pulp exposure in mature permanent teeth. However, it is often an expensive procedure, may require multiple appointments, and requires a high level of training and clinical skill, specifically in molars. Uninsured patients, low-income patients, and patients with limited access to specialist care often elect for extraction of restorable teeth with irreversible pulpitis. There is a need for an alternative affordable treatment option to preserve their teeth and maintain chewing function. A case of pulpotomy using calcium-enriched mixture (CEM) cement in two maxillary molars (#14 and 15) in a healthy 36-year-old patient is presented. Both teeth were diagnosed with symptomatic hyperplastic/irreversible pulpitis. Patient did not have dental insurance, was unable to afford RCT, and refused to extract the teeth. CEM pulpotomy and amalgam build-ups were done as an alternative to extraction. At 2-year recall, both teeth were functional with no signs/symptoms of inflammation/infection. Periapical radiographs and 3D images showed normal PDL around all roots. Pulpotomy with CEM biomaterial might be a viable alternative to tooth extraction for mature permanent teeth with hyperplastic/irreversible pulpitis, and can result in long-term tooth retention and improved oral health.

## Introduction

Root canal therapy (RCT) is the universal treatment for mature permanent teeth with carious pulp exposure and irreversible pulpitis. The outcome of RCT in vital/nonvital teeth has been extensively studied [1]; it is a successful procedure with favorable prognosis [2]. However, this treatment is an expensive procedure, can require multiple appointments, and also requires a high level of training and clinical skill, specifically in molar teeth [3]. Therefore, for uninsured patients, low-income patients, and patients with limited access to specialist care, sometimes the treatment of choice for a molar with irreversible pulpitis is tooth extraction.

An alternative option for these patients who desire to save their teeth is a vital pulp therapy (VPT) procedure [4]. Vital pulp therapy is the treatment of choice following carious pulp exposure in immature teeth [5]; it has a favorable outcome due to adequate pulpal blood supply and the healing potential of pulp tissue [6]. However, studies on long-term outcome of VPT in mature teeth with irreversible pulpitis are limited [7], which makes it challenging to present it as a predictable treatment option to patients.

The outcome of VPT depends on the type of material used to cover the remaining coronal/radicular pulp. Calcium-silicate based cements are the materials of choice. These materials are biocompatible and bioactive. They produce hydroxyl apatite crystals in contact with tissue fluids [8], which gives them hard tissue induction potential and sealing ability when in contact with dentin [9]. Mineral trioxide aggregate (MTA) is the most extensively studied cement in this group [10]. MTA has shown successful clinical results in VPT of immature [5] and mature [11-13] teeth with carious pulp exposure and established irreversible pulpitis. 

Calcium-enriched mixture (CEM) is a tooth-colored cement with internal phosphate reservoir. This cement is bioactive even in the presence of distilled water [14]. Histological studies have shown the biocompatibility, osteogenesis [15] and hard tissue induction potential of CEM cement when used as a pulp capping material [16, 17]. CEM cement has been successfully used in VPT of immature teeth with traumatic pulp exposure [18], immature teeth with carious pulp exposure [5, 19], and mature teeth with carious pulp exposure and clinical symptoms of irreversible pulpitis [20-22]. Previous studies have shown that outcomes of CEM pulpotomy in mature molars with irreversible pulpitis are comparable with outcomes of MTA pulpotomy and of root canal treatment [11, 20]. This case report presents successful CEM pulpotomy in two mature maxillary molars with extensive carious lesions and pulp polyps. CEM pulpotomy was used as an alternative to RCT/extraction in this case. Outcomes are recorded clinically and radiographically (2D and 3D).

## Case Report

**Figure 1:**
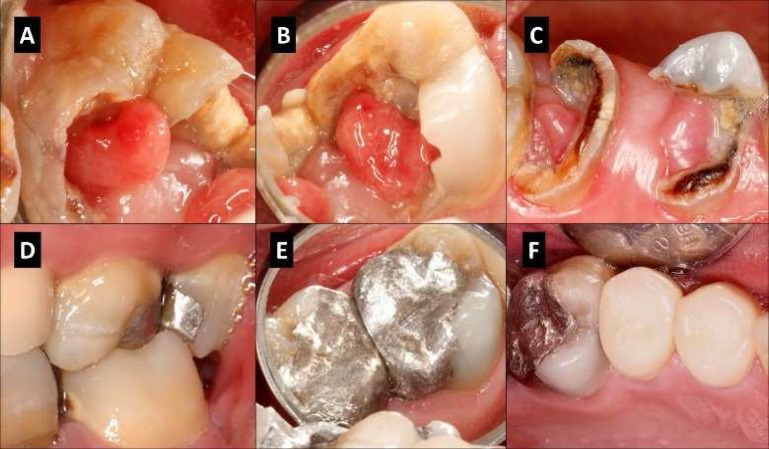
Clinical view of the upper left teeth; *A)* Preoperative occlusal view tooth #15; *B)* Occlusal view tooth #14; *C)* Occlusal view teeth #12 and #13. Note the gingival over-growth, pulp polyps, and the plaque accumulation due to lack of function and appropriate oral hygiene; *D-F)* Clinical images at 2-year recall session

**Figure 2:**
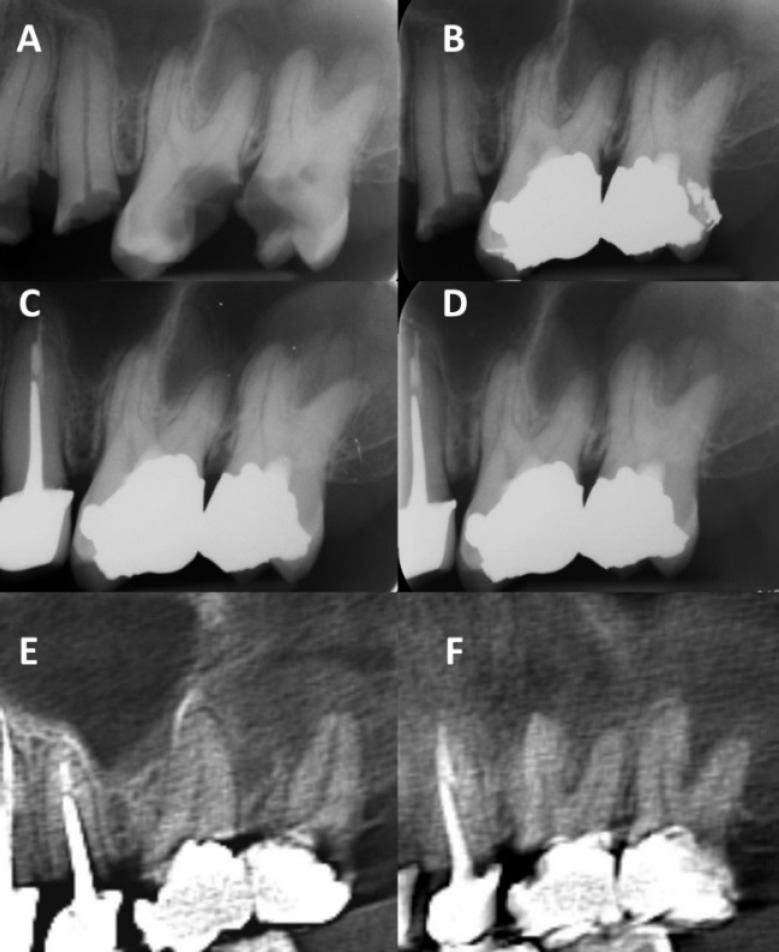
*A)* Preoperative periapical radiograph. Note the extensive caries in molars and normal periodontium on all roots; *B)* Immediate postoperative radiograph; *C)* One-year recall; *D)* Two-year recall. Note the normal PDL space and normal radiographic view of the roots and canals (no internal/external root resorptions); *E)* CBCT view, sagittal section on palatal roots #14 and #15; *F)* CBCT view, sagittal section on buccal roots #14 and #15 showing normal periodontium

A 36-year-old male was referred by his general dentist for evaluation and endodontic treatment of # 12-15. The chief complaint was “I cannot chew on my left side”. The patient described his past hesitation about seeking dental care due to dental anxiety, financial issues, and lack of dental insurance. Patient’s medical history was non-contributory. Teeth #12-15 were severely damaged due to extensive caries. The crowns of the teeth were partially covered by gingival over-growth and pulp polyps (Figure 1A-1C). Teeth #12-15 responded positively to cold test (Roeko, Coltene Whaledent, Langenau, Germany) and were mildly sensitive to percussion. There was no sensitivity to palpation on the apices and no sinus tracts. Pre-operative periapical radiographs showed extensive carious lesions on #12-15. There was no PDL widening and no periapical radiolucencies (Figure 2A). A diagnosis of symptomatic irreversible pulpitis with pulp polyp was made for #12-15. The general dentist planned to do scaling and root planning on #12-15, and offered the patient the following treatment plan options to restore the upper left posterior teeth: 

*i)* Gingivectomy and RCT #12-15; full-coverage crowns #12-15; 

*ii)* Gingivectomy and RCT #12-13, full-coverage crowns #12-13, extraction/implant #14-15; *iii)* Extraction/implant #12-15.

Patient did not have dental insurance and could not afford to pay in full for any of these three treatment plan options. No other less expensive options were offered by the referring dentist. Patient stated that he did not want to extract his maxillary molars and was eager to find a financially affordable solution to save them. Pulpotomy with CEM cement followed by amalgam core build-up was offered to him as an alternative treatment option. Patient chose the CEM pulpotomy option because it was financially affordable and offered him a chance to save his maxillary molars. Written informed consent was obtained. The referring dentist was contacted and he agreed with the treatment plan. The treatment of the premolars is not presented as they are not the focus of this manuscript.

Local anesthesia was obtained using 2% Lidocaine HCL with 1:80,000 epinephrine (Daroupakhsh, Tehran, Iran). CEM pulpotomy was performed on one tooth at a time. After isolation, caries was completely excavated in #14, the pulp chamber was then unroofed and full pulpotomy was performed. Bleeding was not controlled by gently placing a cotton pellet soaked in 2.5% NaOCl on the pulp chamber floor for ~5 min. Therefore, CEM cement powder and liquid (BioniqueDent, Tehran, Iran) was mixed and 3 mm layer of biomaterial was tamponed on the pulp chamber floor and canal orifices. The rubber dam was then removed and a matrix band was placed around the tooth using a Tofflemire holder. Amalgam core build-up restoration was done (SDI gs80, SDI limited, Australia). The procedures were repeated on tooth #15 at the same session. A postoperative radiograph was taken to evaluate the quality of the procedures (Figure 2B). RCTs of the premolars were completed in another session and the patient was referred back to the general dentist for full-coverage crown restorations on #12 and 13.

## Results

Patient was contacted one week later for follow-up. He stated he had discomfort for few days after the procedure but now he could chew using his upper left molars. Recalls were performed one year and two years after the treatment (Figures 1E-1F and 2C-2F). At both recall sessions, teeth #14 and 15 were functional and asymptomatic (no pain on percussion/palpation). Mobility and probing depths for both teeth were within normal limits.

Periapical radiographs showed normal PDL around #14 and 15 at both recall sessions. At the 2-year recall, a cone beam computed tomography (CBCT) scan (Scanora 3D, Soredex company, Helsinki, Finland; FoV 6×6 cm, Voxel size 130μ) of #14 and 15 was taken, which showed no periapical radiolucency and normal PDL spaces around all roots of both teeth (Figure 2E and 2F).

## Discussion

Initial RCT procedures are highly predictable and successful for restorable teeth with irreversible pulpitis [1]; however, there are still numerous patients who choose tooth extraction. Most of these patients are uninsured or low-income, or have limited access to a highly skilled dentist or endodontist. A survey of 1195 dentists showed that 79% reported frequently encountering an endodontic condition, but only 34% reported performing any type of definitive endodontic procedure. Lack of insurance was the greatest barrier to care in north Carolina, with 89% of dentists considering it as a moderate to major barrier, followed by cost of the endodontic treatment (87%) [23]. 

Dental insurance is a strong predictor of the likelihood of seeking preventive dental care. Uninsured children are 2.5 times less likely than insured children to receive dental care in USA [24]. Studies in USA showed that poor and low-income people were less likely to have private dental coverage than were people with higher incomes [25]. Those with incomes at or above the poverty level are twice as likely to report a dental visit in the past 12 months as those who are below the poverty level [24]. Due to these socio-economic realities, many restorable teeth with irreversible pulpitis are being extracted yearly. Further, most of these patients do not have the means to properly replace their missing teeth. A study on the effect of tooth loss on physical and mental health showed that older adults who have significant tooth loss are less functional compared to those who have lost fewer teeth [26]. Pulpotomy is a significantly less expensive and less complicated procedure compared to RCT. Therefore, for patients suffering from irreversible pulpitis who cannot afford RCT, a full pulpotomy might be a realistic alternative treatment option to save their teeth.

Previous studies have evaluated the outcome of VPT in human mature permanent teeth as compared to other treatment modalities. Examination of inclusion/exclusion criteria reveal that some studies only included cases with reversible pulpitis [27, 28]. Therefore, the outcome of these studies might not be applicable to teeth with irreversible pulpitis. A five-year clinical trial of pulpotomy with CEM cement compared to RCT in molar teeth with established irreversible pulpitis showed no significant difference in clinical outcomes [20]. Also, there were no significant differences in outcomes of CEM full pulpotomy compared with MTA pulpotomy in mature molars with irreversible pulpitis [11]. A prospective clinical study on MTA pulpotomy in mature teeth diagnosed with reversible or irreversible pulpitis showed success rate of 92.7% [12]. A systematic review on the outcome of full pulpotomy showed favorable success rate (over 90% at two-year follow-up) in treating carious vital pulp exposures of permanent posterior mature teeth [4]. The study concluded that coronal pulpotomy treatment could increase tooth retention by providing an alternative option particularly for low-income patients or in under-served areas worldwide. The results of our case report corroborates these findings. Furthermore, the presented case shows how an alternative treatment option (CEM pulpotomy/amalgam build-up compared to RCT/full-coverage crown) can still save a patient’s oral health and function, unlike tooth extraction with no realistic option to replace the missing teeth.

The clinical and radiographic criteria for success of pulpotomy in mature teeth with carious pulp exposure differ between studies. Absence of clinical signs/symptoms (including pain on percussion/palpation, sinus tract, deep periodontal pockets) [11, 12, 20, 28] or pulpal responsiveness to vitality tests [28] were defined as clinical success. Absence of internal/external root resorption [11, 12, 20, 27], normal PDL [11, 12, 20, 27, 28], formation of a mineralized bridge under capping material [12, 27, 29] or narrowing of the root canal space [12] were criteria for radiographic success of the treatment. The criteria for success of pulpotomy in mature teeth with carious pulp exposure have been summarized in a recent review study [30]. None of the clinical studies have documented outcomes of pulpotomy in mature teeth with irreversible pulpitis using 3D imaging as we did in this case. 3D imaging is a more sensitive and more accurate diagnostic tool to detect periapical rarefactions compared to digital and conventional periapical radiographs [31, 32]. Uraba *et al.* showed that CBCT imaging is effective at detecting periapical lesions that cannot be detected on periapical radiographs specifically in maxillary molars [32].

The material used for VPT should be biocompatible, induce hard tissue production by pulp, and create a long-lasting seal. Bioactivity is the key factor, which makes calcium silicate-based cements suitable for VPT. CEM cement has shown clinically acceptable long-term sealing ability when used as obturation material [33, 34], or coronal plug in regenerative endodontic treatments [35]. CEM cement has shown hard tissue induction potential when used as pulp capping material [16], and also when used as perforation repair material [33, 36] or root-end filling material [37, 38] adjacent to periodontal tissues. Bioactivity of CEM cement is likely the main reason for its biocompatibility, hard tissue induction potential, and sealing ability.

## Conclusion

In conclusion, pulpotomy with CEM cement might be a viable alternative to tooth extraction for mature molars with hyperplastic/irreversible pulpitis, and can result in long-term tooth retention and improved oral health. CBCT imaging is a sensitive and valuable tool to assess the outcome of VPT in mature teeth.
